# “You Feel Like You Did Something So Wrong”: Women's Experiences of a Loved One's Child Sexual Abuse Material Offending

**DOI:** 10.1177/10778012231208974

**Published:** 2023-10-23

**Authors:** Michael Salter, Delanie Woodlock, Christian Jones

**Affiliations:** School of Social Sciences, 7800 University of New South Wales, Sydney, NSW, Australia

**Keywords:** child sexual abuse, feminism, secondary victims, child sexual abuse material

## Abstract

This article examines the experiences of female partners and relatives of child sexual abuse material offenders and the (il)legibility of their experiences within prevailing theoretical frameworks and policy responses to violence against women. Drawing on survey and interview data with clients of a specialist support agency, we situate the lack of understanding and support available to these women within the systematic depoliticization of child sexual abuse. The article traces how women developed their own social critique of child sexual exploitation as a form of gendered violence and called for a feminist reengagement with the politics of child sexual abuse.

This article examines the experiences of female partners and relatives of child sexual abuse material (CSAM) offenders and the (il)legibility of their experiences within prevailing theoretical frameworks and policy responses to domestic violence. Their experiences are made illegible as they fall between the gaps in knowledge and understanding of both sexual and domestic violence. The article draws on the findings of an evaluation of PartnerSPEAK, an organization in Victoria, Australia, that supports the nonoffending partners, family, and friends of CSAM offenders. Through our analysis of our survey and interviews with PartnerSPEAK clients, we map the absence of community and professional understanding that confronted women as they realized their partner or family member was a CSAM offender. We situate this gap within shifts, over time, in responses to gendered violence in Australia and internationally, in which feminist connections between violence against women and children have given way to the “siloing” of domestic violence and child abuse as discrete areas of theory and practice. In this article, we trace how women navigated the systemic gaps and silences around their experiences as partners and family members of CSAM offenders, and their development of a social and political framework around their experiences through shared dialogue and advocacy. These insights reveal intimate connections between the sexual exploitation of children and violence against women that were recognized in second-wave feminism but have since been overlooked in feminist theory and practice. We argue that these insights offer a contemporary foundation and agenda for a feminist reengagement with the politics of child sexual abuse.

## Feminism and CSAM

CSAM represents a significant and growing form of gender-based violence. The majority of people convicted of CSAM are male and anonymous online surveys suggest that between 2.2% and 4.4% of men have intentionally viewed CSAM (Dombert et al., 2016; Seto et al., 2015). One large content analysis of CSAM found that 80% of victims were girls and 78% were under the age of 12 (C3P, 2016). The most highly traded and in-demand CSAM images depict the abuse of young girls by their fathers (Seto et al., 2018). The long-term impacts of CSAM victimization are considerable, with survivors reporting complex trauma as well as hypervigilance and anxiety about the ongoing distribution of their abuse images (C3P, 2017; Gewirtz-Meydan et al., 2018). Ongoing distribution leaves survivors vulnerable to extortion and harassment from CSAM consumers (C3P, 2017). In the absence of a proactive response from the government or technology industry, some survivors are forced to seek out and report their own images to authorities in an effort to stay safe (Salter & Hanson, 2021). The volume of reports of CSAM to U.S. authorities has increased by 50% per year since 2008 (Bursztein et al., 2019, p. 1), with 21.7 million reports of suspected CSAM made to U.S. authorities in 2020 (each report may contain multiple images or videos; NCMEC, 2021).

Despite the epidemic nature of CSAM and the dire situation of victims and survivors, contemporary feminist engagement with the problem of CSAM has been muted and often reflects a skeptical tone (Bray, 2011). This was not always the case. CSAM was a foundational concern for feminists in the 1970s and 1980s within broader conflicts over the role of male power and gender inequality in child sexual abuse and pornography (e.g., MacKinnon, 1985; Rush, 1980). While some feminist scholars continue to be actively engaged with the problem of child sexual abuse (Campbell, 2023; Nelson, 2016), recognition of children as victims of sexual violence within feminist frameworks has considerably diminished over the last three decades (Hlavka, 2019; Whittier, 2009, 2015). Hanson (2019) is a notable exception to this trend, situating the proliferation of CSAM within the rise of “cyber-libertarianism” as the legitimizing ideology of the technology sector. She suggests that social norms around male sexual entitlement have intersected with the profit motives of technology companies to produce a largely unregulated online environment replete with harmful sexual content and a myriad of opportunities for sexual exploitation. Feminist accounts of intersectionality are poised to make an important contribution to the field (Bernard, 2019).

However, the specific issue of CSAM has been largely neglected in contemporary feminist discourses on sexual violence (Bray, 2011) even as feminist engagement in the role of technology in intimate partner and sexual violence has burgeoned (McGlynn et al., 2017). Bray (2009) argues that the feminist retreat from the issue of child sexual abuse in general, and CSAM in particular, reflects the systematic depoliticization of child sexual abuse. Key to this depoliticization has been the recasting of second-wave feminism concerns about child sexual abuse as a prurient “moral panic” grounded in intolerance and denial of children's “sexual agency” (Bray, 2009). For example, Filippello (2020) situates public backlash against the use of child models in fashion photography within a so-called “social panic” around what the author refers to derisively as “kiddie porn.” In another example, Taylor (2018) positions the criminalization of CSAM as an expression of “carceral feminism,” and argues that the criminalization of CSAM may make CSAM more desirable. Both Filippello (2020) and Taylor (2018) link social and legal prohibitions against CSAM to right-wing and conservative agendas. Such work suggests that the appropriate feminist stance toward CSAM is one of skepticism, critical distance, and potentially, decriminalization.

A diminished feminist engagement with the problem of child sexual abuse is not only evident in intellectual work but also in policy and practice responses. While child sexual abuse was understood by second-wave feminists as part of the “continuum” of men's violence (Kelly, 1988), the professionalization of responses to child sexual abuse has coincided with the medicalization of the problem and its decontextualization from feminist critique and politics (Armstrong, 1994). There is increasing recognition of the cooccurrence and overlap between domestic violence and child maltreatment (Henry, 2018) however the complex relationship between child sexual exploitation and violence against women is underacknowledged in both scholarship and practice (Jones et al., 2021). Women who discover that their male partner has been accessing CSAM are an acute example of a group whose experiences cannot be easily accommodated through such fragmented theoretical or service approaches, and their experiences are examined in more detail in the following section.

## Women Partnered to CSAM Offenders

A large number of CSAM offenders have partners and families. Arrest data for CSAM in the 2010–2011 financial year in Australia, New Zealand, Italy, and the United States found that 42% of offenders were cohabitating with a partner or children and 31% were found to be living with their parents or grandparents (Bouhours & Broadhurst, 2011, p. 9). Between 21% and 65% of CSAM offenders in treatment have a partner and 25–47% have at least one child (Brown & Bricknell, 2018). Rates of (known) contact offenses by men with CSAM convictions vary from 10% to 38% (Brown & Bricknell, 2018). Taken together, these findings provide evidence that a high number of partners and families are impacted by men's CSAM offending.

The partners and families of CSAM offenders are described by Walker (2019) as secondary victims, due to the profound impacts of discovering their loved one's offending on their lives and well-being. In one of the only interview studies conducted with this cohort, Liddell and Taylor (2015) interviewed nine Australian women who had partners who were CSAM offenders. Women spoke of the judgment they received from their families, friends, and the wider community, and they were often seen to be complicit in, or a cause of, their partner’s CSAM use. They detailed significant mental health impacts and isolation, with their lives ruptured by the discovery of their partner's CSAM offending. Women's shock and confusion were often compounded by the lack of social support and information, with many finding the police and court processes confusing and retraumatizing. These findings dovetail with broader research into the families of sex offenders, who report high rates of stigmatization, social isolation, and shame (Bailey & Klein, 2018; Duncan et al., 2020; Kilmer & Leon, 2017; Levenson & Tewksbury, 2009). Mothers in particular have been stigmatized by the offending of their partners and were historically held responsible for the sexual offenses of their partners (Azzopardi et al., 2018).

As CSAM convictions increase exponentially, this mother-blaming narrative is being replaced by policy frameworks with an emphasis on the protective role that partners can play in preventing further offending (Duncan et al., 2020). A range of research studies has positioned nonoffending partners as resources for the facilitation of desistance and prevention of recidivism through the supervision and monitoring of their partner's behavior and safeguarding their children from further abuse (Shannon et al., 2013). While the overt mother-blaming narrative may have lessened, there is now seen to be an exploitative burden placed on mothers to ameliorate the risk of child sexual offending by their partners (Duncan et al., 2020). The needs and experiences of nonoffending partners and family are rarely considered separately from those of the perpetrator or children, despite the available research indicating that they face significant trauma and upheaval in the aftermath of discovering the CSAM offending of their loved one (Duncan et al., 2020; Liddell & Taylor, 2015; Stitt, 2007; Wager et al., 2015).

Elsewhere, we have argued that CSAM offending in a relationship often occurs in the context of coercive control and domestic violence (Jones et al., 2021). We found that women's experiences leaving a relationship with a CSAM offender have many parallels with the trajectories of women leaving domestically violent and controlling relationships (Jones et al., 2021). Other research with women partnered to male sex offenders finds relationships characterized by male dominance and aggression (Iffland et al., 2016). These findings only underscore the importance of a feminist perspective on the experience and rights of women whose partners use CSAM, however, their specific experiences fall into a significant gap within feminist theory and state responses to family and domestic violence and image-based sexual abuse. It is in this gap that PartnerSPEAK clients emerge, and our study explores their experiences in a vacuum of understanding and support. We were guided in our analysis by the research question: What are the lived experiences of nonoffending partners and family of CSAM offenders?

## Methodology

This article presents a secondary analysis of PartnerSPEAK program evaluation data, which consisted of a survey of 53 PartnerSPEAK clients and in-depth interviews with seven clients (Jones et al., 2021). All survey and interviewee respondents were over 18 years of age. The evaluation approach was developed in collaboration between the authors and PartnerSPEAK's senior management team to ensure the appropriateness of the methodology given the uniqueness and complexity of PartnerSPEAK's mission and client group. The survey was circulated to clients through the PartnerSPEAK online forum and social media accounts, and survey respondents were given the option of volunteering for an online interview. The survey and interviews were focused on participant’s experiences of PartnerSPEAK services and their broader support needs relating to their partner's or family member's offending.

Qualitative survey responses were analyzed in Qualtrics. Qualitative survey responses and interviews were transcribed and NVivo was used in the coding of the transcripts. Survey and interview data collection and analysis occurred simultaneously. We applied the system of thematic analysis as outlined by King and Horrocks (2010), which we detail below.

### Survey

The article is based on a sample of 45 responses. The survey received 53 responses, including 38 who completed the entire survey, 4 who completed 95% of the survey, and 4 who completed 51–55% of the survey. Eight participants answered less than one-third of the questions and were excluded from the analysis. The survey included 33 multiple choice questions gathering data on demographics, involvement, and satisfaction with PartnerSPEAK and their unmet support needs. Participants had the option of three open-text questions, in which they could expand on their support needs, share any other information that they had not been asked about, and to provide contact details if they wanted to be interviewed. Survey demographic information is displayed at Table 1.

**Table 1.** Survey Demographic Information.

Demographic	Results	Number	Percentage
Gender	Female	43	95.6
	Male	1	2.2
	Did not specify	1	2.2
Sexual orientation	Heterosexual	40	88.9
	Lesbian	0	0
	Bisexual	5	11.1
Language spoken at home	English	44	97.8
	Other	1	2.2
Aboriginal and Torres Strait Islander	Yes	4	8.9
	No	40	88.9
	Did not specify	1	2.2
Place of residence	Victoria	16	35.6
	New South Wales	7	15.6
	South Australia	7	15.6
	Queensland	1	2.2
	West Australia	2	4.4
	Overseas	1	2.2
Age	18–24	2	4.4
	25–34	3	6.7
	35–44	17	37.8
	45–54	12	26.7
	55–64	8	17.8
	65+	2	4.4
Living with a disability	Yes	5	11.1
	No	40	88.9
Relationship status	Single	6	13.3
	De facto/married	12	26.7
	Separated/divorced	25	55.6
	Widowed	2	4.4
Minor children at home	Yes	25	55.6
	No	20	44.4

### Interview

Seven PartnerSPEAK clients indicated on the survey that they would like to be interviewed. They were interviewed via Zoom teleconferencing software from July to August 2020 by the second author. An interview guide was used to ensure consistency across interviews. The questions were focused on their experiences with PartnerSPEAK as well as the context of how they came to seek support through the service. An example of a question asked was “What prompted you to seek support from PartnerSPEAK?” Six of the women were partnered to the male offender, and one was the daughter of a male offender.

### Ethical Considerations

The project was approved by the UNSW Human Research Ethics Committee (HC No. 200295). All survey and interview participants were provided with a detailed information sheet about the project prior to their participation and had the opportunity to contact the research team to address any questions. Participants had the option of being interviewed by a female researcher, and all opted for this. The information sheet sent to the participants prior to the interview included details about the research, examples of questions asked, and the option to withdraw their consent throughout the process. Participants were not remunerated for their contributions. The identities of all survey and interview participants were unknown to PartnerSPEAK. Survey participation was anonymous, and the confidentiality of the interviews was assured through the deidentification of transcripts, in which direct identifiers (such as names) and indirect identifiers (such as other information that, when combined, could identify the participant) were removed. While the study involved questions about difficult personal experiences, all participants were in contact with PartnerSPEAK and thus had access to a specialist support service, although they were also provided with contact information about other support services as an alternative.

### Template Analysis

The overall findings of the evaluation indicated that there was a connection between other forms of violence against women, such as domestic violence perpetration, and CSAM offending. The findings also showed that there was a culture of silence around these connections, and the experiences of nonoffending partners of CSAM offenders in general. We recoded the qualitative data using Template Analysis, which is a form of thematic analysis that involves developing a coding template that can include themes that have been identified before the coding, as well as themes that evolve through the process of coding (King & Horrocks, 2010). This method of analysis was suited to our research as we had previously identified themes in our first overall coding of the evaluation and we were now recoding the data to gain further insights.

Interview transcripts were recoded and thematically analyzed using the three-step process detailed by King and Horrocks (2010). The first step was to code the transcripts descriptively, this included codes such as “police coming to the door.” The next step was to code interpretively, in which we generated three main interpretive codes, which were common across the interview sample:

1. Discovery: How the women discovered their loved ones offending and their discovery about CSAM in general;
2. Aftermath (which includes their trauma, grief, impacts on children, friends and family, and legal implications. Also, their doubts about themselves, their sexuality, and having a relationship with someone who is a sex offender); and
3. Connections (this includes connections with other forms of abuse, such as domestic violence, as well as connections with other women, and broader social issues).

This process of recoding and thematic reorganization was undertaken iteratively within the research team, with regular meetings to discuss emerging findings. The final stage of thematic analysis is to define an overarching theme, which is a response to the overall research question of “what are the lived experiences of the nonoffending partners and families of CSMA offenders?” which we identified as “silence and isolation.” This silence and isolation unfolded against a backdrop where there is a lack of structural analysis to assist women to make sense of what has happened to them and their children. These overarching interview themes are presented below supplemented by survey data to offer additional empirical evidence.

## Findings

Here we present the three interpretive thematic findings, mapping women's experiences of discovering that their partner was using CSAM, the aftermath, and then their efforts to make sense and meaning out of their experiences. These efforts involved forming connections between their experiences and other forms of gender-based violence, as well as developing relationships with other women and key services. In doing so, we argue, women are forging new conceptual and practice frameworks for the harms committed against them; harms that are currently invisible and unrecognized within contemporary theory and policy arrangements.

### Discovery

The traumatic discovery of their partners’ or family members’ CSAM use was a ubiquitous theme among all interviewees. Discovery typically involved a succession of revelatory events rather than a single moment. For 87.8% (*n* = 40) of survey participants, the CSAM offending had been reported to police. For three of the seven women interviewed, they were first notified that their partner was accessing CSAM during a police search of their house, a shocking and bewildering event that catalyzed an unfolding process as they uncovered the extent of their partner's deception and betrayal. Client interviewee 7 described her bewilderment when confronted at home by police with a search warrant, and the indescribable “despair” as she realized their purpose:

The police knocked on the door and I had a migraine, I’d just come out of the hospital. They knocked on the door and the kids went: “There's police at the door”. I’m like “oh, what do they want? let them in”. I was thinking maybe someone's car got stolen—you just presume something else. Then they came in and said there's a warrant out for the house to be searched. And you’re going “pardon, what?” You’re in despair. You can’t describe it ….

The execution of search warrants usually occurred around dinnertime, and this mix of the mundane and traumatic could create a sense of surrealness to the event. Everyday activities such as cooking meals became intermingled with wholly unexpected and previously unthinkable discoveries. Interviewee 5 explained:

We had the officers at the door because they had set up a sting or whatever, so he had gone out with the intention of—it wasn’t just the online, a large part was also acting on. So when they arrived at my door I was cooking dinner and they came in and I could be present when they spoke with my daughter. We still get very emotional, she gets quite confused by the whole experience. But it was just a very overwhelming experience. I didn’t want them to go anywhere near her, then when they showed me who they were, it was a surreal experience. We can never eat chicken kebabs again!

When police execute a search warrant for CSAM offenses, they cannot assume that the partner is unknowing or nonoffending, given the range of recent convictions demonstrating that some women have colluded in their partner's CSAM offending in various ways (Salter et al., 2021). However, initial suspicions that they may be involved in their partner's offenses only amplified the fear and bewilderment that women experienced during police investigation. For interviewee 4, the CSAM offender was her father, who she did not live with. When the police came to her parents’ house with a search warrant, her mother, who is a lawyer, insisted that police follow due process and refused to cooperate in implicating her husband in wrongdoing. As a result, her mother also felt that suspicion fell upon her. Interviewee 4 said:

They executed a search warrant on him and that's when it became clear the extent of his pornography addiction. That's how everyone found out. It was still a shock … It's kind of completely outside the realm of anything you would consider to happen.

So the feeling of real complete kind of confusion. The way I’d describe it is a really fast de-anchoring, feeling really adrift and not knowing really where to go, what to do, what's happening ….

My mum said she found when she was not cooperating in trying to implicate him the police kind of switched to “well, you knew about this”, trying to imply that she’d been involved in some kind of illegal activity, which she hasn’t.

The sense of “deanchoring” and feeling “adrift” was common throughout women's experiences. Interviewee 1 discovered her husband's offending when she walked in on him directing others to abuse a child via the internet. She said: “When I walked in and he was engaged with live online sex, which means he's telling these pricks what to do to this kid.” She characterized this moment as instantly catastrophic. She felt that she was “tough” but was unsure how she could overcome the “mountain” of abuse and lies:

This is psychologically catastrophic, how am I going to survive this? Because I knew the state I was in. I had fallen to the bottom of a fucking mountain going “I have to climb back up this fucking mountain”. I’m 60, how am I going to do this, emotionally and mentally and physically? I thought, for the first time in my life, I was facing something that I genuinely thought I don’t know if I’m going to survive. It is not merely your world's just fallen apart. It is as if you have lost your internal organs, you can’t breathe.

This evocative description of falling off a mountain and having to climb back up at 60 years of age captures the sense of profound injury and isolation experienced by women in our study. For interviewee 1, surviving the discovery of her partner's child abuse offending is a matter of her own individual resources—emotional, mental and physical—and yet she feels disemboweled and hollowed out, even unable to breathe. This description is a vivid illustration of the particular ways that this crime invades women's sense of security and attacks their core sense of self.

Like interviewee 1, interviewee 6 also discovered her husband's offending herself. There had been no police involvement, which was relatively uncommon in the research study. Only 8.9% (*n* = 4) of survey respondents indicated that CSAM offending had not been reported to police. Interviewee 6 works in the domestic violence sector and consulted with forensic professionals about the material she found, who concluded that this material would not be seen as criminal. She explained how she initially found the CSAM:

I found a USB in the study one day while he was at work that was obviously hidden, and initially, I had a laugh to myself. I was like, “oh”… because he's always said he's not a user of pornography or anything like that. So I had a bit of a giggle and thought, “oh yeah, sure you’re not!” And I didn’t initially look at it because I didn’t have any concerns. But it got the better of me and I had a look at it later that day and found the material. I was in shock. I called him and told him what I’d just found. That's initially what I did.

When asked if she took the CSAM to the police she said:

What I found were images and those images were just of children, not in a sexual way. But then other images of say close-up sexual acts where you couldn’t identify if it was a child or not. But I obviously know what that was used for, so I sought the opinion of people that I knew that had worked in forensics with sex offenders and things like that and they felt the same, that it wouldn’t be chargeable.

As interviewee 6 did not feel she could bring the evidence to the police, she had little option but to share custody of her children with the offender after they separated. This was a common dilemma amongst women interviewed for this study and will be discussed next in more detail (Table 2).

### Aftermath

The consequences of the discovery of their loved ones CSAM offending were vast and long-lasting for the participants in our research. Table 2 details the wide-ranging impacts on participants. The serious impacts of discovering the CSAM offending of a partner or a loved one could not be disentangled from the lack of support and acknowledgment encountered by women. Throughout the interview with interviewee 1, she repeatedly stated that there was “no pamphlet” to support her through this process; that is to say, there was no resource or agency she could turn to. She didn’t know what to do, who to speak to, or even how to articulate this catastrophic moment in her life:

There's no pamphlet. There are no rules. There's no fucking pamphlet. What is that? You fall down, I’ll start CPR. There's a car accident, you know who to call! Who the fuck do you call with this shit?! And no one wants to know.

For interviewee 2, the process of comprehending her ex-partner's betrayal was further complicated by the fact that her husband had sexually abused their daughter. She explained:

**Table 2.** Impacts From Discovering a Loved Ones CSAM Offending

Impacts	Number	Percentage
Mental health	41	91.1
Commenced or increased anxiety or depression medication	21	46.7
Increased reliance on alcohol, nicotine or other drugs	10	22.2
Trust in future relationships	33	73.3
Relationships with family	28	62.2
Relationships with friends	24	53.3
Parenting affected	25	55.6
Changes to their relationship with their children	21	46.7
Changes to their relationships with their grandchildren	4	8.9
Negative impacts on their physical health	25	55.6
Financial wellbeing	30	66.7
Housing	21	46.7
Employment	16	35.6
Reputation impacted	15	33.3
Cyber-bullying and harassment	2	4.4

It was everything, everything—where do I live, what do I do, how do I deal with people, where do I put this? Is there shame, is there blame? There are lots of things that initially you don’t even think of for yourself because you’re so worried about your children and where you’re going to live and what you’re going to do.

This sense of having no internal framework of understanding, or social process to move through, was exacerbated by the privacy and secrecy of court processes. Interviewee 7 expressed frustration that, throughout the investigation and prosecution process, privacy laws prevented police from telling her the “true story” of her ex-partner's offending. For privacy reasons, some women were not informed about the kinds of rehabilitation their ex-partner underwent while incarcerated, however, they were required to facilitate his access to their children when he was released. Interviewee 5 detailed this dilemma:

[H]ow do you deal with that relationship when in law they’re meant to have that meaningful relationship [between her child and her ex-partner]—how do you define that, how do you define what sort of meaning they would get?

Because I’m finding when I do speak with counsellors there's that “oh, mistakes have been made, but the research shows that the value of that parent is far greater than the damage” you know? It's like, but my child when she's older will turn around and say to me “you failed to protect me”.

Surely there's a balance. It should be mandatory supervision. The other parent should know the psychiatric status of that person, what's been the rehabilitation? We should have access to that information if they want access to their child.

This quote highlights several intersecting issues for the women who were mothers in this research. Firstly, they are left to grapple with the reality that the father of their children accessed CSAM, and the implications of his offending for his parenting. As interviewee 5 asks, what is a “meaningful relationship” between a child and a father who is sexually aroused by images and videos of children being abused? Secondly, women's concerns about their children, and their own feelings about the offender, can be set aside in a family law context that privileges shared care between parents, even where one parent has been convicted of child sex offences. Thirdly, while the mother does not ultimately determine the outcomes of these decisions and processes, she feels responsible for them, and anticipates being held accountable by her children. Finally, mothers are being asked to make critical decisions without full information about the risk posed by the offender but will likely bear any adverse consequences visited upon their children due to enforced contact with their father.

For interviewee 3, once the court process was finalized, she encountered a lack of clear advice and consistent directives regarding her ex-partner's permissible contact with their children. She was required by the court to provide him with unsupervised access to them, and he was permitted to live with the children of his new partner, but she was threatened with the removal of her children if she left her children to stay with him for a period of time. She explained:

There were no clear directions at all about the contact and what was allowed to occur and what wasn’t allowed to occur. I had to phone child protection on my own to find out because I was going overseas and the perpetrator was having contact unsupervised, but would I be allowed to leave my child there or what? And then they became so aggressive to me, child protection, saying they would remove my child if I was to leave him with the perpetrator. And I said “[H]e's living with other children!”

Women described navigating day-to-day parenting at the intersection of simultaneous criminal justice, child protection and family law matters, while facing the significant financial and practical implications of separation, divorce and the incarceration of their ex-partner. Interviewee 5 details the ripple of destabilization that occurred as a result of her partner's CSAM offending. She said:

My main concerns when all that happened was just a high level of anxiety. I was struggling to deal with everything—take the kids to school, trying to explain why the police wanted to speak with my daughter—all those things were very overwhelming. Just dealing with the deception as well, like not understanding what the bail conditions were, for instance. I wasn’t allowed to know until human services came along and explained what my rights were. The loss of home, we lost our home. Just trying to steer them through that and family wanting to know what was happening and not being able to talk about anything with anyone.

While women often lacked important information, advice and support, they could be restricted from explaining their situation to friends and family due to court and legal requirements, and also due to the shame of the offending. They spoke in vivid terms of their stigmatized status. Interviewee 1 said that, after her partner's conviction, she was treated as a “car wreck,” “mental,” “damaged,” “not really worthy of actually being listened to or heard.” Interviewee 7 said that she couldn’t speak to friends and family about her circumstances because “they will judge you.” Interviewee 5 described her ex-partner's offending as a shameful “shadow” that still follows her. The shadow of their loved ones offending left many women questioning if they were somehow complicit in his crimes, and how they could have missed such a core aspect of their partners’ life. Interviewee 1 said: “The very first stand I took was, what has been my role in this? What has been my role? Hearing that you’ve been used or that you’ve missed this somehow, I had to work out how that's happened.” Interviewee 5 described “the shame, my personal anger with myself for not knowing” and trying to navigate that “It wasn’t your fault, even though you feel like you did something so wrong by… you were enabling in some way or through my ignorance or not knowing.”

This self-doubt was exacerbated as they realized the full extent of their ex-partner's deception. Interviewee 5 recounted her dawning realization that she did not really know her husband. She said “…being in court and listening to a completely different assessment of his psychological makeup and thinking this has probably been a very long-term integral part of that person's profile. All the deception and manipulation.” For interviewee 7, she felt she could not trust being in a relationship with another man, and that she lost trust in most people's relationships as a result. She explained:

Because of the morals, I’ve been brought up and the topic, you can’t believe that. Like how can anybody be attracted to children? It's mind-boggling, you can’t…Then you lose trust. Even in another relationship you go towards or any friends or anyone you meet, and you say “what have you been doing today” and they go “I’ve been on the computer”, I automatically think they’re looking up something bad. I can’t help not to think that. Anybody in any relationship or if they’re getting married, you think how do you know he's not looking it up? How do you know?

Women's experiences of the aftermath of discovering their loved one's CSAM offending were shaped by systemic but also epistemic gaps and absences. Not only were criminal justice and child protection services poorly attuned to their needs, but also they often had to shoulder the responsibility of rebuilding their own, and their children's, lives in the absence of social supports and specialist services. They typically had no frame of reference through which to make sense of their experience. Indeed, it was this vacuum and absence of understanding that drove many of them to connect their experiences to broader contexts of violence against women, and the social and political silences around CSAM offending, as we explore in the next section.

### Connection

Women found the silence and lack of understanding surrounding CSAM offending and its impacts intolerable. They abhorred the epistemic vacuum in which their experiences drifted in the absence of frameworks of action and meaning. Interviewee 1 responded with fury to the disinterest and passivity that she felt obscured the prevalence and seriousness of CSAM offending. She questioned the absence of social protest and concern, and linked that absence to permissible environments for offenders:

Who else gets it?! Because if they got it, they would be fuckin’ marching up and down the fucking street? When a woman is raped, and we “Reclaim the Night”. We need to be far more public about this crime. There's no campaigning on TV. The only thing I can think is because it's a crime believed by most people to be within the family. Well, guess what? It isn’t. I don’t get people's complacency with it. I think, is it just me? Have I missed something? Like, have I missed something or are people just pretending it doesn’t happen. This has happened because there's a systemic and perpetual silence around this crime. And it works for the criminals.

This connection to a “systemic and perpetual silence” around CSAM offending was also mentioned by other women. Interviewees expressed bewilderment about professional and social responses to CSAM. As they overcame the initial shock, some women expressed intense curiosity about the causes of pedophilia and motives for CSAM offending. They were looking for scientific explanations for their ex-partner's behavior, but they were often left wanting. Interviewee 7 said:

There's no explanation why they’re like this. What's wrong with their brains? I don’t have any answers. You got to a psychologist and they say “it's chemically imbalanced, what was his childhood like?” I’m going, “What's a childhood got to do with why would you want to look that up?”

Interviewee 4 called for an investigation into the “overall world” of CSAM, including the predominance of male perpetration. She criticized a narrow focus on criminal justice responses and the apparent lack of academic interest in the broader contexts of CSAM offending. She said:

There seems to be an attitude that the way CSAM offending is handled through the police and justice system is the only way that this phenomenon, as dark as it is, can be handled. There's no exploration of—and I think that can feed into the notion that they can’t be rehabilitated, they can’t change attitude. I don’t think there's much exploration or coverage of academia or any kind of investigation of the overall world of this kind of crime.

These quotes foreground the ways in which women began to construct a *political* and *social* perspective on CSAM and responses to it, grounded in their own experiences, reflections, and inquiry. They criticized the secrecy and social silencing surrounding the crime, as well as individualized psychological and criminal justice responses, and instead called for CSAM to be addressed as a social problem and shared crisis. They made links between their partner's offending and other forms of gendered violence, such as sexual assault and domestic violence. One-fifth of survey (*n* = 9) respondents indicated contact with domestic violence services, and a number of interviewees described patterns of control, aggression, and violence by their ex-partner as well as his CSAM offending. However, these links were not present in the responses they received from government systems and services. It is notable that some of the women who were interviewed about their partner's CSAM offending were employed in the domestic and family violence sector. When asked if there was any awareness or connections made in their workplaces with CSAM and domestic violence, they all said no, and that they had not felt comfortable raising these issues. However, the relationship between CSAM offending and other forms of gendered violence were often clear to women in their own experience. 20% (*n* = 9) of survey participants reported that they had been in contact with domestic and family violence services. Interviewee 6 reported that her partner was not only a CSAM offender but also a domestic violence perpetrator. She said:

I guess looking back there was always a little bit of emotional, psychological violence. I guess that sort of started becoming more prevalent, I guess I was probably in a better headspace to acknowledge it and respond better to it. But it came to a head when he assaulted me last year. I’d already said that I wanted to separate.

Financial abuse and control around money was noted by several women as one of the key ways that they were controlled by the CSAM offender. Interviewee 1 said that her fear of opening her own bank account revealed to her the depth of her partner's control over her. She explained:

I did not know how controlling a marriage I was in until I went to try and open a bank account. The fear and the terror of opening a—I was hysterical. I was in the car just screaming in fear. And you go, where is this fear coming from? I don’t understand. But it's years and years—it's like a boa constrictor just slowly taking off your oxygen more and more.

She continued that she had always felt fear in her relationship, but did not feel like her experiences were domestic violence:

I can remember 15 years ago I heard on the radio someone saying if you feel like you’re walking on eggshells you’re in an abusive relationship. And I thought, shit, I feel that! But for the life of me, I didn’t know what it was.

Her husband controlled her use of technology also, and one of her key messages to women whose partners were CSAM offenders was: “The first thing you need to do is to get another phone! Your safety and everything else after that is dependent on you having a phone that they don’t have control over.” Women spoke of not knowing where they fitted in the violence against women sector and being unaware of services that could assist them. Interviewee 5 explained:

It was the police officer who referred me to services because I had no idea that the PartnerSPEAK service was available. And I couldn’t define where we fitted in in terms of domestic abuse and things like that, and with the children.

Other women connected the impacts of having a loved one being a CSAM offender with their history of being a victim of child sexual abuse. Interviewee 7's stepfather sexually abused her when she was a child. She recently reported his crimes and he was arrested and charged. Due to this she was able to access victims of crime compensation and used this for mental health support. However, she explained that there was no support for how the CSAM offending had impacted her, and no validation of her trauma. She said:

Now with what I’ve been through here, I’ve had to pay for my own psychology. The police say we’ll help you, what do you need help with? But they don’t help you at all. They won’t even talk to you. No one will help. If you’ve gone on antidepressants, you’re still paying for everything. Yet we’ve—oh, how do I say it? What I went through with my stepfather, I was a little bit compensated, a tiny bit. I had a little bit of justice, a little bit of—he got arrested. Otherwise, he got away with it. And I’m not asking for food vouchers or anything, but…

Interviewer: You need that validation that you’re a victim of this as well.

Exactly.

While women often made the connections to wider issues of men's violence against women and children, they recognized there was a lack of collective advocacy in creating awareness of CSAM. This led to several of them feeling that they had a personal responsibility to keep fighting for the children that their loved one was involved in harming. Interviewee 1 said:

The only reason I will continue to pursue this is because there are still kids being harmed. I can’t live with myself and know what that man is still continuing to do, and engage with kids like that. Because I want to die knowing that I’ve done every fucking thing I can do.

## Discussion and Conclusion

This article has detailed the way that women's experiences of a loved one's CSAM offending are rendered illegible because these experiences fall between what is recognized as sexual and domestic violence. However, as the findings are based on a secondary analysis of a small sample of interviews and surveys gathered as part of an evaluation of one support service, the findings of the study are not generalizable to all nonoffending family, partners, and ex-partners of CSAM offenders. The majority of respondents in our study left the relationship with the offender once they discovered the offending, which perhaps reflects the broader culture of PartnerSPEAK (Jones et al., 2021). However, UK research with nine nonoffending partners of sex offenders found that only one separated from the offender, which suggests that women have diverse responses to a partner's CSAM offending (Duncan et al., 2020). There is a need for research with larger, representative samples of adults regarding their concerns and responses to a loved one's known or suspected CSAM offending, as well as a need for research into CSAM offending in the context of more diverse relationships contexts, including LGBTIQ+ and culturally diverse relationships.

Our study builds on other evaluations of support options for families of sex offenders, which consistently highlights the efficacy of self-help and peer support approaches in promoting coping strategies and resilience (Sample et al., 2018; Kavanagh & Levenson, 2021). However, this article has emphasized the politicized awareness that emerged from women's contact with each other and PartnerSPEAK. Liddell and Taylor (2015) previously documented women's multiple and profound losses due to a loved one's CSAM offending, and these findings have been underscored by similar research in the United Kingdom (Duncan et al., 2020). Although in our study, through shared dialogue, interviewees forged political critiques and demands that connected their lived experience to structures of gendered inequality and capital accumulation. These critiques mirrored in many ways the insights of those feminist scholars who have paid attention to the expanding black market in CSAM, such as Bray (2009, 2011), who foregrounded the links between CSAM, violence against women, and the political economy of the technology industry.

However, the expanded political horizon of the women in our study came at a considerable cost to them, and often followed months or years of crisis, trauma, and systemic maltreatment. It was apparent that none of the women interviewed had substantive knowledge of the problem of CSAM before they discovered their loved one's offending, reflecting broader social silences and denial of the issue. The discovery of their loved one's offending was characterized by bewilderment and loss, as they sought to make sense out of an unfolding set of revelations that led them into areas of life shrouded in secrecy and shame. There was no cognitive schema or support process to structure and validate women's responses to this unprecedented upheaval and irrevocable rupture in their lives.

In the absence of accessible frameworks of meaning or action, the revelation of their loved one's offending confronted women with basic existential questions. When interviewee 2 asked rhetorically, “Where do I put this?,” she was pointing to the fact that her experiences no longer had a comprehensible social place or location. Women's prior sense of self and identity, and their capacity to trust themselves or others, came under sustained challenge as they realized the extent of their loved one's betrayal, and the infectious nature of the stigma of their offending. Women described themselves as diminished in the eyes of their family, friends, and communities. This sense of diminishment and alienation was amplified within systems that were experienced as cold and insensitive to their specific needs. Elsewhere, we have proposed that CSAM offending in a relationship can be understood as a form of family and domestic violence (Jones et al., 2022), however, the lack of recognition of CSAM offending in the service system introduced additional complexities and uncertainties. When a father is a CSAM offender, what is in the “best interests” of their child postseparation? What is “fathering” in such a context? As investigations and prosecutions for CSAM increase each year, the population of impacted family and friends also grows, foregrounding the need for professionals and services to acknowledge and develop a comprehensive response to the challenges that CSAM consumption poses to family arrangements and the protection of children related to the offender.

Inevitably, women found their own answers. In an interview, they named the invisibility and silence that surrounded their loved one's crimes and that shrouded their own experiences in shame. They recognized that this silence not only contributed to their own trauma and distress but that it facilitated the ongoing abuse and exploitation of children. They asked consequential questions about their loved one's offending and their sexual interest in children and expressed dissatisfaction with the simplistic explanations offered by their psychologists. Interviewee 4 called for inquiry into the “overall world” of CSAM offending beyond the narrow forensic focus of the criminal justice system. Indeed, in women's own experiences, links between the “overall world” of CSAM offending and broader patterns of violence against women and children were evident, although the connections between child sexual exploitation and broader patterns of gendered violence were largely unacknowledged within the myriad of systems and services that women found themselves entangled within. Our findings suggest that feminist reengagement with child sexual abuse as a social and political issue, and the development of holistic support and advocacy frameworks, would further enhance existing theory and practice in the field of domestic violence, and the lived experience and reflections of the ex-partners of CSAM offenders offers important insights into the multiple connections between child sexual exploitation and violence against women.

## References

[bibr1-10778012231208974] Armstrong L. (1994). Rocking the cradle of sexual politics: What happened when women said incest. Addison-Wesley.

[bibr2-10778012231208974] Azzopardi C. Alaggia R. Fallon B. (2018). From Freud to feminism: Gendered constructions of blame across theories of child sexual abuse. Journal of Child Sexual Abuse, 27(3), 254–275. 10.1080/10538712.2017.1390717 29161221

[bibr3-10778012231208974] Bailey D. J. Klein J. L. (2018). Ashamed and alone: Comparing offender and family member experiences with the sex offender registry. Criminal Justice Review, 43(4), 440–457. 10.1177/0734016818756486

[bibr4-10778012231208974] Bernard C. (2019). Using an intersectional lens to examine the child sexual exploitation of black adolescents. In Pearce J. (Ed.), Child sexual exploitation: Why theory matters (pp. 193–208). Policy Press.

[bibr5-10778012231208974] Bouhours B. Broadhurst R. (2011). Virtual global taskforce P2P online offender sample, July 2010–June 2011. Australian National University.

[bibr6-10778012231208974] Bray A. (2009). Governing the gaze: Child sexual abuse moral panics and the post-feminist blind spot. Feminist Media Studies, 9(2), 173–191. 10.1080/14680770902814835

[bibr7-10778012231208974] Bray A. (2011). Merciless doctrines: Child pornography, censorship, and late capitalism. Signs, 37(1), 133–158. 10.1086/660178

[bibr8-10778012231208974] Brown R. Bricknell S. (2018). *What is the profile of child exploitation material offenders? Trends and Issues in Crime and Criminal Justice, Australian Institute of Criminology*. https://www.aic.gov.au/publications/tandi/tandi564.

[bibr9-10778012231208974] Bursztein E. Clarke E. DeLaune M. Elifff D. M. Hsu N. Olson L. Shehan J. Thakur M. Thomas K. Bright T. (2019). *Rethinking the detection of child sexual abuse imagery on the internet*. Paper presented at the World Wide Web Conference. https://dl.acm.org/doi/pdf/10.1145/3308558.3313482.

[bibr10-10778012231208974] C3P. (2016). Child sexual abuse images on the internet. Canadian Centre for Child Protection. https://protectchildren.ca/en/resources-research/child-sexual-abuse-images-report/ .

[bibr11-10778012231208974] C3P (2017). Survivor's survey preliminary report. Canadian Centre for Child Protection. https://protectchildren.ca/en/resources-research/survivors-survey-results/ .

[bibr12-10778012231208974] Campbell B. (2023). Secrets and silence: Child sex abuse from Cleveland to Savile and beyond. Policy Press.

[bibr13-10778012231208974] Dombert B. Schmidt A. F. Banse R. Briken P. Hoyer J. Neutze J. Osterheider M. (2016). How common is men's self-reported sexual interest in prepubescent children? Journal of Sex Research, 53(2), 214–223. 10.1080/00224499.2015.1020108 26241201

[bibr14-10778012231208974] Duncan K. Wakeham A. Winder B. Armitage R. Roberts L. Blagden N. (2020). The experiences of non-offending partners of individuals who have committed sexual offences: Recommendations for practitioners and stakeholders. Nottingham Trent University & University of Huddersfield. https://huddersfield.box.com/s/1sumdnyq9yjkgwhw0axzvgt7e2rfgcih .

[bibr15-10778012231208974] Filippello R. (2020). Eccentric feelings: Little girls’ pleasures on the feminist fashion set. Australian Feminist Studies, 35(105), 217–238. 10.1080/08164649.2020.1843998

[bibr16-10778012231208974] Gewirtz-Meydan A. Walsh W. Wolak J. Finkelhor D. (2018). The complex experience of child pornography survivors. Child Abuse & Neglect, 80, 238–248. 10.1016/j.chiabu.2018.03.031 29631255

[bibr17-10778012231208974] Hanson E. (2019). Losing track of morality’: Understanding online forces and dynamics conducive to child sexual exploitation. In Pearce J. (Ed.), Child sexual exploitation: Why theory matters (pp. 87–116). Policy Press.

[bibr18-10778012231208974] Henry C. (2018). Exposure to domestic violence as abuse and neglect: Constructions of child maltreatment in daily practice. Child Abuse & Neglect, 86, 79–88. 10.1016/j.chiabu.2018.08.018 30273814

[bibr19-10778012231208974] Hlavka H. R. (2019). Regulating bodies: Children and sexual violence. Violence Against Women, 25(16), 1956–1979. 10.1177/1077801219875817 31718531

[bibr20-10778012231208974] Iffland J. Berner W. Dekker A. Briken P. (2016). What keeps them together? Insights into sex offender couples using qualitative content analyses. Journal of Sex & Marital Therapy, 42(6), 534–551. 10.1080/0092623x.2015.1079757 26280194

[bibr21-10778012231208974] Jones C. Salter M. Woodlock D. (2022). Someone who has been in my shoes”: The effectiveness of a peer support model for providing support to partners, family and friends of child sexual abuse material offenders. Victims & Offenders, 18(4), 715–731. 10.1080/15564886.2022.2051108

[bibr22-10778012231208974] Jones C. Woodlock D. Salter M. (2021). *Evaluation of PartnerSPEAK.* UNSW. https://www.academia.edu/60971032/Evaluation_of_PartnerSPEAK.

[bibr23-10778012231208974] Kavanagh S. Levenson J. (2021). Supporting those who love the loathed: Trauma-informed support groups for family members of registered sex offenders. Families in Society: The Journal of Contemporary Social Services, 103(2), 208–220. 10.1177/10443894211008846

[bibr24-10778012231208974] Kelly L. (1988). Surviving Sexual Violence. Blackwell.

[bibr25-10778012231208974] Kilmer A. Leon C. S. (2017). Nobody worries about our children’: Unseen impacts of sex offender registration on families with school-age children and implications for desistance. Criminal Justice Studies, 30(2), 181–201. 10.1080/1478601x.2017.1299852

[bibr26-10778012231208974] King N. Horrocks C. (2010). Interviews in qualitative research. Sage.

[bibr27-10778012231208974] Levenson J. Tewksbury R. (2009). Collateral damage: Family members of registered sex offenders. American Journal of Criminal Justice, 34(1-2), 54–68. 10.1007/s12103-008-9055-x

[bibr28-10778012231208974] Liddell M. Taylor S. C. (2015). Women's experiences of learning about the involvement of a partner possessing child abuse material in Australia. PartnerSPEAK.

[bibr29-10778012231208974] MacKinnon C. A. (1985). Pornography, civil rights, and speech. Harvard Civil Rights-Civil Liberties Law Review, 20(1), 1–70.

[bibr30-10778012231208974] McGlynn C. Rackley E. Houghton R. (2017). Beyond ‘revenge porn’: The continuum of image-based sexual abuse. Feminist Legal Studies, 25, 25–46. 10.1007/s10691-017-9343-2

[bibr31-10778012231208974] NCMEC. (2021). *2020 reports by electronic service providers*. National Center for Missing and Exploited Children. https://www.missingkids.org/content/dam/missingkids/gethelp/2020-reports-by-esp.pdf.

[bibr32-10778012231208974] Nelson S. (2016). Tackling child sexual abuse: Radical approaches to prevention, protection and support. Policy Press.

[bibr33-10778012231208974] Rush F. (1980). The best kept secret: Sexual abuse of children. McGraw Hill.

[bibr34-10778012231208974] Salter M. Hanson E. (2021). I need you all to understand how pervasive this issue is”: User efforts to regulate child sexual offending on social media. In Bailey J. Flynn A. ,, & Henry N. (Eds.), The Emerald international handbook of technology facilitated violence and abuse (pp. 729–748). Emerald Publishing.

[bibr35-10778012231208974] Salter M. Wong W. T. Breckenridge J. Scott S. Cooper S. Peleg N. (2021). Production and distribution of child sexual abuse material by parental figures. Australian Institute of Criminology. https://doi.org/10.52922/ti04916

[bibr36-10778012231208974] Sample L. L. Cooley B. N. Ten Bensel T. (2018). Beyond circles of support: “fearless”—an open peer-to-peer mutual support group for sex offense registrants and their family members. International Journal of Offender Therapy and Comparative Criminology, 62(13), 4257–4277. 10.1177/0306624(18758895 29478390

[bibr37-10778012231208974] Seto M. Buckman C. Dwyer R. Quayle E. (2018). Production and active trading of child sexual exploitation images depicting identified victims. NCMEC & Thorn. https://www.missingkids.org/content/dam/missingkids/pdfs/ncmec-analysis/Production%20and%20Active%20Trading%20of%20CSAM_FullReport_FINAL.pdf .

[bibr38-10778012231208974] Seto M. Hermann C. A. Kjellgren C. Priebe G. Svedin C. G. Långström N. (2015). Viewing child pornography: Prevalence and correlates in a representative community sample of young Swedish men. Archives of Sexual Behavior, 44(1), 67–79. 10.1007/s10508-013-0244-4 24515803

[bibr39-10778012231208974] Shannon K. L. Pearce E. Swarbrick R. (2013). Factors influencing the development of an innovative service for women non-offending partners (NOPs) of male sexual offenders. Journal of Sexual Aggression, 19(3), 357–368. 10.1080/13552600.2012.729092

[bibr40-10778012231208974] Stitt S. (2007). Non-offending mothers of sexually abused children: The hidden victims. The ITB Journal, 8(1), 13-37. doi:10.21427/D7016T

[bibr41-10778012231208974] Taylor C. (2018). Anti-carceral feminism and sexual assault—A defense. Social Philosophy Today, 34, 29–49. 10.5840/socphiltoday201862656

[bibr42-10778012231208974] Wager N. M. Wager A. R. Wilson C. (2015). Circles South East’s program for non-offending partners of child sex offenders: A preliminary outcome evaluation. Probation Journal, 62(4), 357–373. 10.1177/0264550515600541

[bibr43-10778012231208974] Walker N. (2019). Intentional peer support as a trauma informed response to the partners of perpetrators of child sexual abuse material. Churchill Trust, https://www.churchilltrust.com.au/fellow/natalie-walker-vic-2017/ .

[bibr44-10778012231208974] Whittier N. (2009). The Politics of Child Sexual Abuse: Emotion, Social Movements, and the State. Oxford University Press.

[bibr45-10778012231208974] Whittier N. (2015). Where are the children? Gender & Society, 30(1), 95–108. 10.1177/0891243215612412

